# Involvement of Hypoxia-Inducible Factor-1 in the Inflammatory Responses of Human LAD2 Mast Cells and Basophils

**DOI:** 10.1371/journal.pone.0034259

**Published:** 2012-03-28

**Authors:** Vadim V. Sumbayev, Inna Yasinska, Abraham E. Oniku, Claire L. Streatfield, Bernhard F. Gibbs

**Affiliations:** Medway School of Pharmacy, University of Kent, Chatham Maritime, United Kingdom; National Institute of Environmental Health Sciences, United States of America

## Abstract

We recently showed that hypoxia-inducible factor 1 (HIF-1) plays a crucial role in the pro-allergic functions of human basophils by transcriptional control of energy metabolism *via* glycolysis as well as directly triggering expression of the angiogenic cytokine vascular endothelium growth factor (VEGF). Here, we investigated HIF-1 involvement in controlling the synthesis of angiogenic and inflammatory cytokines from various human effector cells stimulated by IgE-dependent or innate immune triggers. Purified primary human basophils, LAD2 human mast cells and THP-1 human myeloid cells were used for investigations of FcεRI and Toll-like receptor (TLR) ligand-induced responses. In contrast to basophils, LAD2 mast cells expressed background levels of HIF-1α, which was largely independent of the effects of stem cell factor (SCF). Both mast cells and basophils expressed TLR2 and 4, albeit weakly compared to THP-1 cells. Cytokine production in mast cells following TLR ligand stimulation was markedly reduced by HIF-1α knockdown in LAD2 mast cells. In contrast, although HIF-1 is involved in IgE-mediated IL-4 secretion from basophils, it is not clearly induced by peptidoglycan (PGN). HIF-1α accumulation is critical for sustaining human allergic effector cell survival and function. This transcription complex facilitates generation of both pro-angiogenic and inflammatory cytokines in mast cells but has a differential role in basophil stimulation comparing IgE-dependent triggering with innate immune stimuli.

## Introduction

Human allergic and inflammatory reactions are associated with the activation of effector cells in a ligand-receptor dependent manner [1], [2]. Mast cells and basophils are key effectors of allergic inflammation whereas other myeloid cells, such as monocytes/macrophages and neutrophils, mediate pathogen-induced host innate immune reactions [3]. Allergic inflammation is governed by high-affinity IgE receptor (FcεRI)-IgE-allergen complexes leading to histamine release and production of pro-inflammatory cytokines/eicosanoids [1], [2]. Conversely, pathogen-induced inflammatory responses are triggered *via* pathogen-associated molecular patterns detected by pro-inflammatory Toll-like receptors (TLRs) that induce the expression of inflammatory cytokines in effector cells.

All the above inflammatory responses require effector cells to adapt to stress induced by pro-inflammatory stimulation. The adaptation process is controlled by a number of mechanisms, where the crucial one is activation of the hypoxia-inducible factor 1 (HIF-1) transcription complex [4]–[6]. This complex plays a pivotal role in cellular adaptation to low oxygen availability and to inflammatory stress [4]. HIF-1 is a heterodimeric transcription complex containing the constitutive beta and an inducible alpha subunit, accumulation of which determines HIF-1 transcriptional activity [4]. HIF-1 has a number of target genes (over 40) that control angiogenesis, cell adhesion and glycolysis [4], [5].

We recently reported that the pro-allergic (IgE-mediated) responses of primary human basophils, as well as their capacity to generate the angiogenic cytokine VEGF, involve HIF-1 activation [6]. Different kinds of inflammatory stress, such as pathogen-associated molecular pattern-induced TLR-mediated triggering, were also found to activate HIF-1 in THP-1 monocytes [7]–[8]. In all cases HIF-1 directly controlled VEGF expression on the transcriptional level and facilitated pro-inflammatory cytokine expressions by upregulating glycolysis (thus controlling intracellular ATP levels and preventing their depletion). However, HIF-1 accumulation in THP-1 cells and basophils was governed by differential intracellular signalling mechanisms [6]–[8].

Mast cells and basophils not only express FcεRI receptors but also several TLRs (in particular, TLR 2 and TLR 4 that recognise molecular patterns shared mostly by bacterial pathogens [9]–[13]). Thus, in case of an infection in an asthmatic airway mast cells and basophils could be stimulated by pathogen-associated molecular patterns through TLRs and by IgE-dependent mechanisms [9]–[13]. Monocytes/macrophages, on the other hand, which are usually regarded as key effectors of host innate immune defences, also express FcεRII (CD23) and thereby can participate in allergic responses too [14]. Therefore, these cell types seem capable of contributing to both innate immune and allergic responses. However, it is unclear whether these inflammatory receptors could simultaneously react to both IgE-dependent stimuli and TLR ligands leading to dual inflammatory stress, thus affecting -for example by potentiation- respective types of inflammatory reactions. It is also unclear whether HIF-1 accumulation and modulation of pro-inflammatory cytokine generation differs between human basophils and mast cells. Given the above, in the present study we addressed the role of HIF-1 in human mast cell responses to IgE- and TLR-mediated triggering in comparison to human basophils and THP-1 monocytes.

## Materials and Methods

### Materials

Anti-human IgE, RPMI-1640 medium, foetal calf serum and supplements, DOTAP transfection reagent, enhanced avian HS RT-PCR kit, GenElute™ mammalian total RNA miniprep kit, LPS, PGN, Pam3Cys and HIF-1α-specific siRNA were purchased from Sigma (Suffolk, UK). Maxisorp™ microtitre plates were obtained from Nunc (Roskilde, Denmark). ELISA-based assay kits for detection of VEGF, TNF-α and IL-6, as well as a caspase 3 assay kit, were bought from R&D Systems (Abingdon, UK). IL-4 ELISA kits were purchased from Biolegend (Cambridge BioScience Ltd, UK). Mouse monoclonal antibody to HIF-1α, mouse monoclonal antibody to β-actin as well as rabbit polyclonal HRP-labelled antibody to mouse IgG were from Abcam (Cambridge, UK). Human polyclonal IgE was purchased from Amsbio (Abingdon, UK). Stem-Pro-34 serum-free media and stem cell factor (SCF) were obtained from Invitrogen (Paisley, UK). Quantitative real-time PCR kit and real-time PCR plates were bought from Roche (Burgess Hill, UK). All other chemicals were of the highest grade of purity and commercially available (obtained from Sigma (Suffolk, UK)).

### LAD2 mast cells

LAD2 mast cells were kindly provided by A. Kirshenbaum and D. Metcalfe (NIH, USA) [15]. Cells were cultured in the Stem-Pro-34 serum-free media in the presence of 100 ng/ml SCF.

### Primary human basophils

Human basophils were obtained from buffy coats acquired from healthy blood donors undergoing routine blood donation following ethical approval. This research was approved by the NHS-REC (ref. number: 07/Q1206/3), Blood samples were purchased from the UK National Health Service (NHS) Blood and Transplant (NHSBT) service and were collected in accordance with their internal regulations. Basophils were purified by Ficoll density centrifugation followed by negative selection using magnetic cell sorting [16]. Basophils (94±2% pure, as determined by alcian blue staining with viability of >95%) were incubated for 15 min at 37°C in HEPES-buffered Tyrode's solution and treated as indicated. Following stimulation, histamine release analysis and cell lysis was performed as previously described [6].

### THP-1 human myeloid cells and HEK293 cells

THP-1 human leukaemia monocytic macrophages were purchased from the European collection of Cell Cultures (Salisbury, UK). Cells were grown in RPMI 1640 media supplemented with 10% foetal calf serum, penicillin (50 IU/ml) and streptomycin sulphate (50 µg/ml). HEK293 cells were kept at the same conditions.

### Transfer of HIF-1α siRNA to LAD2 mast cells

HIF-1α-specific siRNA, together with its mutated form (which was used as an inactive control), was obtained from Sigma. Transfection into LAD2 cells was performed using DOTAP reagent [8], [17] according to the manufacturer's protocol. As we previously reported [18], mutated siRNA did not impact the investigated processes (data not shown), confirming that the effects observed were caused by knocking down specific HIF-1α when respective specific siRNAs were used. siRNA was transfected into LAD2 cells using DOTAP transfection reagent according to the manufacturer's protocol. The efficiency of HIF-1α knockdown was 47±7%.

### Western blot analysis

HIF-1α, TLR2 and TLR4 were determined by Western blot analysis and compared to β-actin expressions to assess equal loading, as described in our previous publication [8]. Li-Cor (Lincoln, Nebraska USA) goat secondary antibodies conjugated with fluorescent dyes for 60 min were employed according to the manufacturer's protocol and proteins visualized using a Li-Cor Odyssey imaging system. The Western blot data were subjected to quantitative analysis using Odyssey software and values were normalised against respective actin bands.

### Measurement of HIF-1α and VEGF by quantitative real-time reverse transcription PCR (qRT-PCR)

Total RNA was isolated using GenElute™ mammalian total RNA miniprep kit, followed by HIF-1α mRNA reverse transcriptase–polymerase chain reaction (RT-PCR) performed in accordance with the manufacturer's protocol. Then quantitative real-time PCR was performed. Primer selection was as follows: HIF-1α, 5′-CTCAAAGTCGGACAGCCTCA-3′, 5′-CCCTGCAGTAGGTTTCTGCT-3′ VEGF, 5′-GTATAAGTCCTGGAGCGT-3′, 5′-CTCGGAGGGAGTCCCAAA-3′; actin, 5′-TGACGGGGTCACCCACACTGTGCCCATCTA-3′, 5′-CTAGAAGCATT-TGCGGTCGACGATGGAGGG-3′ [8]. Reactions were performed using a LightCycler® 480 real-time PCR system and respective SYBR Green I Master kit (Roche, Burgess Hill, UK). Analysis was performed according to the manufacturer's protocol. Values representing VEGF and HIF-1α mRNA levels were normalised against those of actin.

### Measurement of TNF-α, VEGF, IL-4 and IL-6 production

Production of TNF-α, VEGF, IL-4 and IL-6 by the cells was analysed by ELISA according to the manufacturer's protocols.

### Histamine release

Mast cell and basophil histamine releases were assessed using spectrofluorometic autoanalysis as previously described [6], [19]. Percentage histamine releases were calculated from the total histamine content in the sum of lysed cell pellet and supernatant vials [6], [19].

### Detection of intracellular ATP

This was analysed using a luminometric kit (Sigma) according to the manufacturer's protocol.

### Measurement of Caspase 3 activity

The activity of caspase 3 was assayed by a colorimetric method based on the hydrolysis of the peptide substrate acetyl-Asp-Glu-Val-Asp-p-nitroanilide (Ac-DEVD-pNA) and carried out according to the manufacturer's protocol.

### MTS cell viability assay

Cell viability was analysed using the Promega (Southampton, UK) 3-(4,5-dimethylthiazol-2-yl)-5-(3-carboxymethoxyphenyl)-2-(4-sulfophenyl)-2H-tetrazolium (MTS) cell viability assay kit according to the manufacturer's protocol.

### In-cell TLR2 and TLR4 assays

This was performed as recently described [20], [21]. Briefly, cells were centrifuged for 5 min at 1200 rpm, washed with respective media and exposed to 2 µg/ml anti-TLR2 or anti-TLR4 antibody for 2 h. This was followed by centrifugation for 5 min at 1200 rpm and washing with media. Cells were then incubated for 2 h with fluorescent dye-labelled Li-Cor (Cambridge, UK) secondary antibodies. Following centrifugation/washing, the cells were transferred into wells of a 96-well plate which was scanned by the Li-Cor Odyssey imaging system. Results were quantified using Odyssey software.

### Statistical analysis

Each experiment was performed at least three times and statistical analysis was conducted using two-tailed Student's t test. Statistical probabilities (p) were expressed as *, where p<0.01.

## Results

### LAD2 mast cells and primary human basophils express TLRs 2 and 4

**Figure 1:**
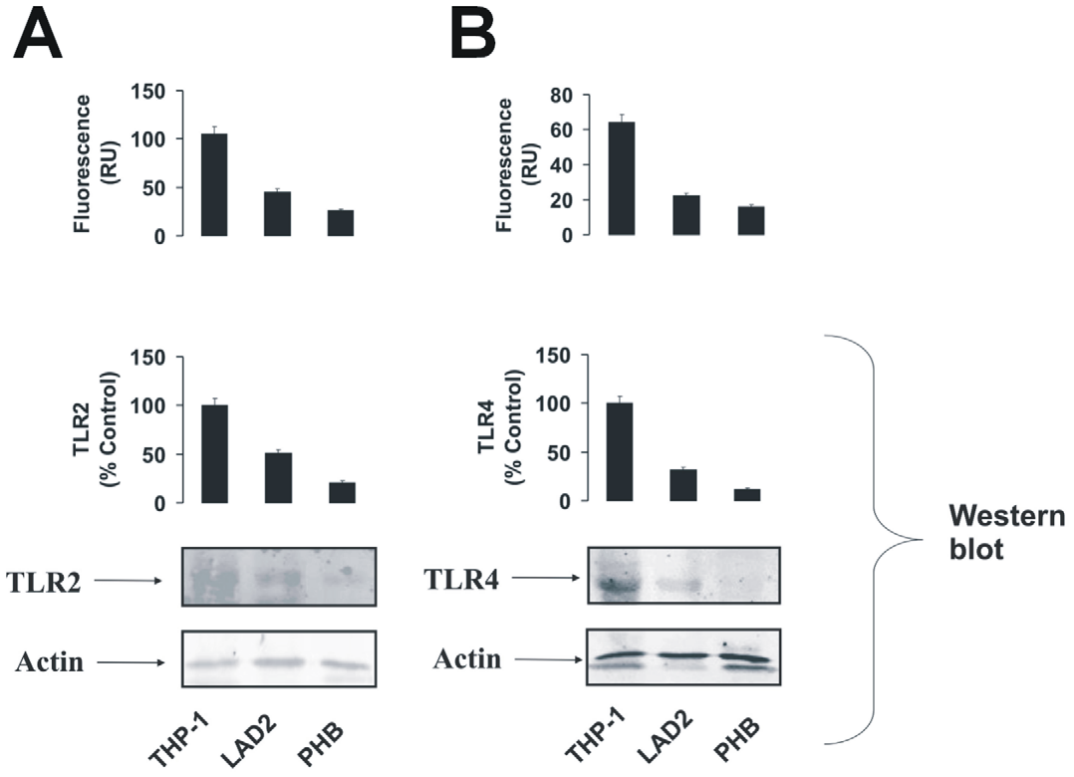
LAD2 human mast cells and primary human basophils express detectable amounts of TLRs 2 and 4. LAD2 cells, primary human basophils (PHB) and THP-1 cells (positive control) were subjected to in-cell TLR2 (**A**) and TLR4 (**B**) assays (upper panel). TLRs 2 (**A**) and 4 (**B**) were also detected in the cell lysates (lower panel). Quantitative data are mean values+S.D. of at least three individual experiments. * indicates p<0.01 vs. control. All Western blot data shown are from one representative experiment out of three that gave similar results.

Human allergic effector cells (mast cells and basophils) are known to produce detectable levels of TLRs 2 and 4 mRNA [9], [10]. Translation of these mRNAs into functional plasma membrane-associated receptors is, however, still questionable. We therefore studied whether LAD2 human mast cells and primary human basophils express TLR2/4 proteins. To analyse this we used Western blot analysis and in-cell TLR2/4 assays. THP-1 human myeloid cells were used as a positive control as they are known to express functional forms of both TLRs [7], [22]. We found that both mast cells and basophils expressed detectable amounts of TLRs 2 and 4, although these expression levels were significantly lower compared to those observed in THP-1 cells (Figure 1).

### LAD2 mast cells accumulate HIF-1α protein which is not upregulated in a TLR2/4-dependent manner

**Figure 2:**
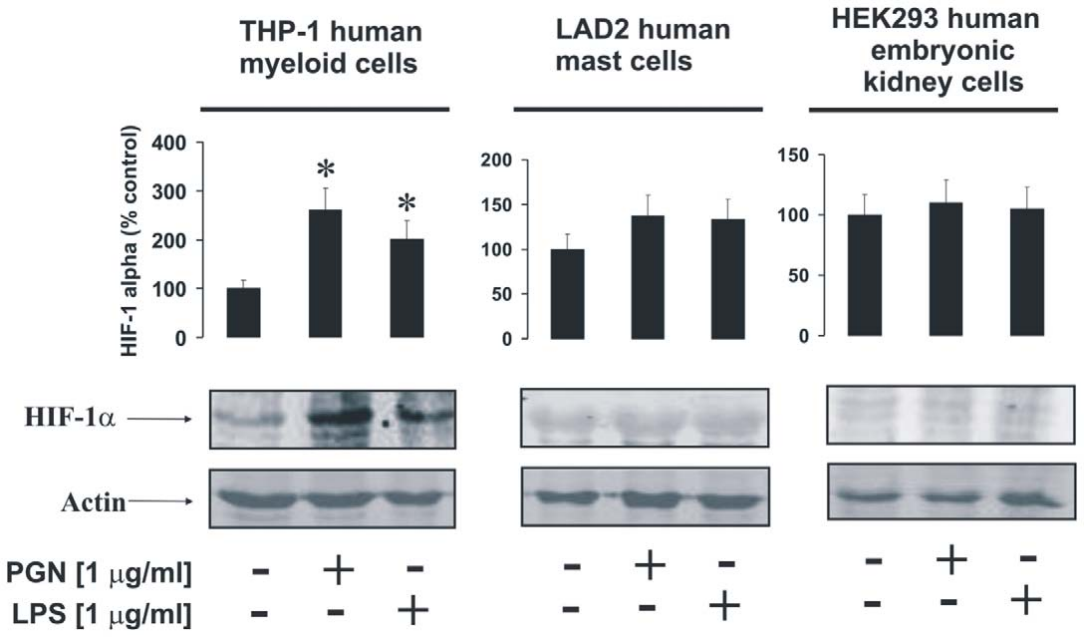
PGN and LPS induce HIF-1α accumulation in THP-1 but not in LAD2 cells. THP-1, LAD2 and HEK293 (negative control – lacking TLR2/4 expression) cells were exposed for 4 h to 1 µg/ml PGN or LPS. HIF-1α accumulation was then analysed. Quantitative data are mean values+S.D. of at least three individual experiments. * indicates p<0.01 vs. control. All Western blot data shown are from one representative experiment out of three that gave similar results.

Recent evidence has demonstrated involvement of the HIF-1 transcription complex in TLR-mediated inflammatory reactions [5]–[8]. We therefore asked whether this case is applicable to LAD2 human mast cells. In THP-1 human myeloid cells we found that 4 h of exposure to 1 µg/ml PGN or LPS led to a significant increase in HIF-1α accumulation (Figure 2). Similar experiments performed with LAD2 mast cells demonstrated HIF-1α accumulation in non-treated cells. Exposure to TLR2/4 ligands did not change the intracellular levels of stabilised HIF-1α protein (Figure 2). HEK293 cells were used as a negative control (they do not express TLRs at all and we have not detected any expression of TLRs 2 and 4 in these cells by in-cell assay and Western blot – data not shown) and did not respond to stimulation with TLR2/4 ligands (Figure 2).

### PGN/Pam3Cys and LPS do not potentiate IgE-mediated pro-allergic responses of LAD2 human mast cells

**Figure 3:**
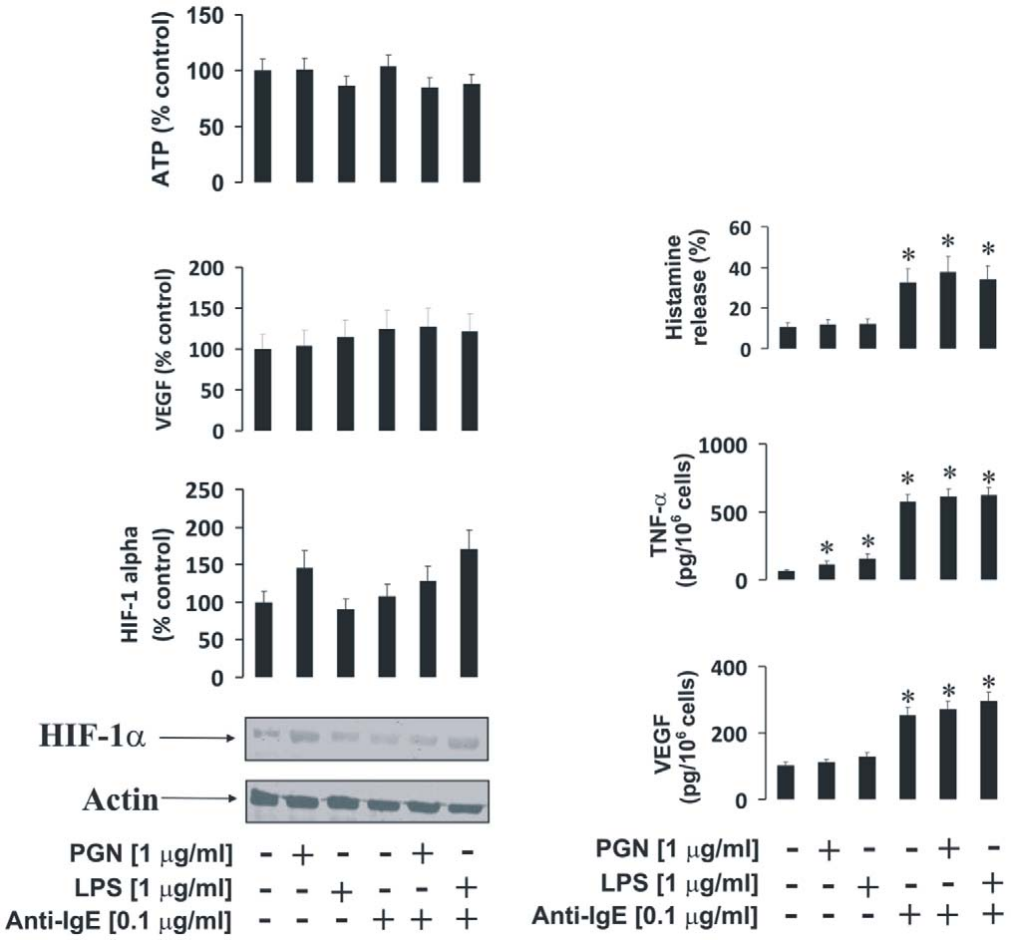
PGN and LPS do not potentiate IgE-induced responses of LAD2 mast cells. LAD2 cells were sensitised with 100 ng/ml IgE for 24 h followed by 4 h exposure to 1 µg/ml PGN or LPS. Thereafter, some samples (including TLR ligand treated and untreated) were exposed to 0.1 µg/ml anti-IgE for 2 h. HIF-1α accumulation, VEGF mRNA expression/VEGF release, histamine and TNF-α release as well as intracellular ATP levels were analysed as described in Materials and methods. Quantitative data are mean values+S.D. of at least three individual experiments. * indicates p<0.01 vs. control. All Western blot data are from one experiment representative of three that gave similar results.

**Figure 4:**
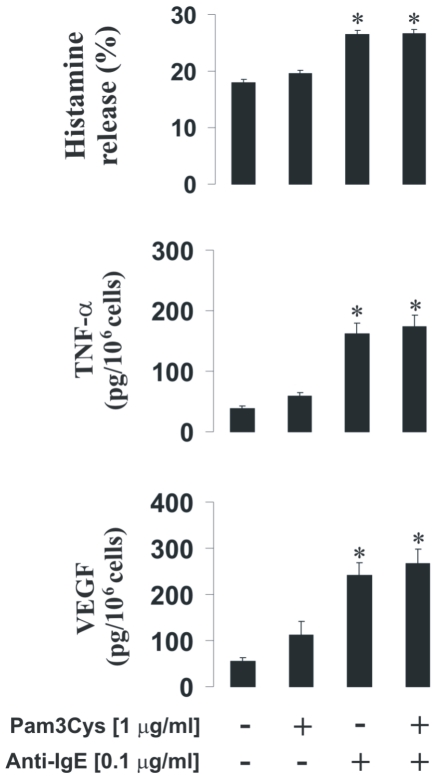
Pam3Cys does not potentiate Anti-IgE response of LAD2 human mast cells. LAD2 cells were sensitised with 100 ng/ml IgE for 24 h followed by 4 h exposure to 1 µg/ml Pam3Cys. Thereafter, some samples (including TLR ligand treated and untreated) were exposed to 0.1 µg/ml anti-IgE for 2 h. Histamine, VEGF and TNF-α release was analysed as described in Materials and methods. Quantitative data are mean values+S.D. of at least three individual experiments. * indicates p<0.01 vs. control.

We investigated whether TLRs 2 and 4 induce pro-inflammatory responses in LAD2 human mast cells. We were also interested whether TLR2/4 ligands potentiate IgE-mediated pro-allergic responses in LAD2 mast cells. Cells were therefore sensitised with 100 ng/ml IgE for 24 h followed by 4 h exposure to 1 µg/ml PGN or LPS (some of the samples were left untreated). Some of the cell samples (including TLR ligand treated and untreated) were then exposed to 0.1 µg/ml anti-IgE for 2 h. We observed that neither TLR2/4 ligands nor anti-IgE alone induced further upregulation of constitutive HIF-1α accumulation. Co-stimulation with PGN or LPS and anti-IgE did not lead to any HIF-1α upregulation either (Figure 3). The same effect was observed in the case of the amounts of the VEGF mRNA (Figure 3). Intracellular ATP levels were not significantly affected, possibly because of basic HIF-1α accumulation in mast cells. TLR2/4 ligands on their own did not induce histamine release and were unable to potentiate IgE-mediated release of this amine. The same effect was observed in the case of VEGF release (Figure 3). Interestingly, both PGN and LPS were able to induce TNF-α production but much lower quantities were released compared to the cases when LAD2 cells were stimulated with anti-IgE in the absence of PGN/LPS. Neither PGN nor LPS were able to significantly potentiate anti-IgE-induced TNF-α production in LAD2 cells (Figure 3). No detectable amounts of IL-4 were released in any case by LAD2 mast cells (data not shown). Since it was reported that PGN could display reduced affinity of TLR2 [23] we controlled our observation by performing exactly the same treatments of LAD2 cells with the alternative TLR2 ligand – Pam3Cys and analysed TNF-α/VEGF as well as histamine release. The results were, however, essentially similar to those observed for PGN (Figure 4).

**Figure 5:**
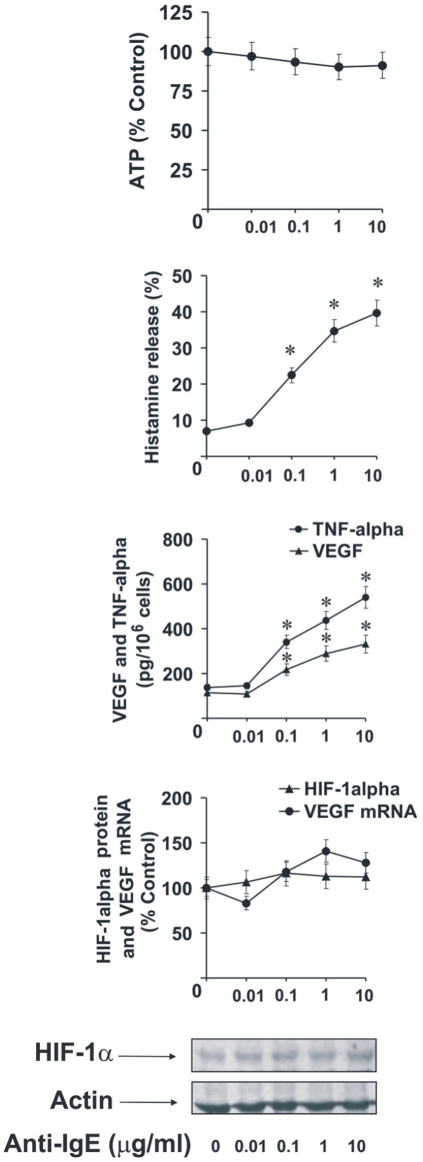
Anti-IgE dose-dependent responses of LAD2 human mast cells. LAD2 human mast cells were sensitised with 100 ng/ml IgE for 24 h followed by 2 h of exposure to increasing concentrations of anti-IgE. HIF-1α accumulation, VEGF mRNA expression/VEGF release, histamine and TNF-α release as well as intracellular ATP levels were analysed as described in Materials and methods. Quantitative data are mean values ± S.D. of at least three individual experiments. * indicates p<0.01 vs. control. All Western blot data are from one experiment representative of three that gave similar results.

Increasing concentrations of anti-IgE were able to upregulate TNF-α/VEGF and histamine release in LAD2 cells after 2 h stimulation. But they were unable to significantly upregulate HIF-1α accumulation and the levels of VEGF mRNA suggesting that the existing basic expression of these two factors may be sufficient to cover the respective functional requirements of the LAD2 cells (Figure 5). No detectable amounts of IL-4 were released in any case by LAD2 mast cells (data not shown).

### Accumulation of HIF-1α protein in non-stimulated LAD2 cells under normal oxygen availability does not depend on SCF

Since SCF was reported to significantly upregulate HIF-1α stabilisation in different cell types [24], we asked whether basic accumulation of this protein in LAD2 cells could be a result of SCF-dependent priming effect. We therefore examined the impact of SCF withdrawal on LAD2 cells that were either non-sensitised or sensitised with 100 ng/ml IgE for 24 h in comparison to LAD2 cells that were cultured in the presence (as normally) of 100 ng/ml SCF and either IgE-sensitized for 24 h or not. Some of the IgE-sensitised cells were then exposed to 0.1 µg/ml anti-IgE for 2 h. HIF-1α accumulation was then analysed.

**Figure 6:**
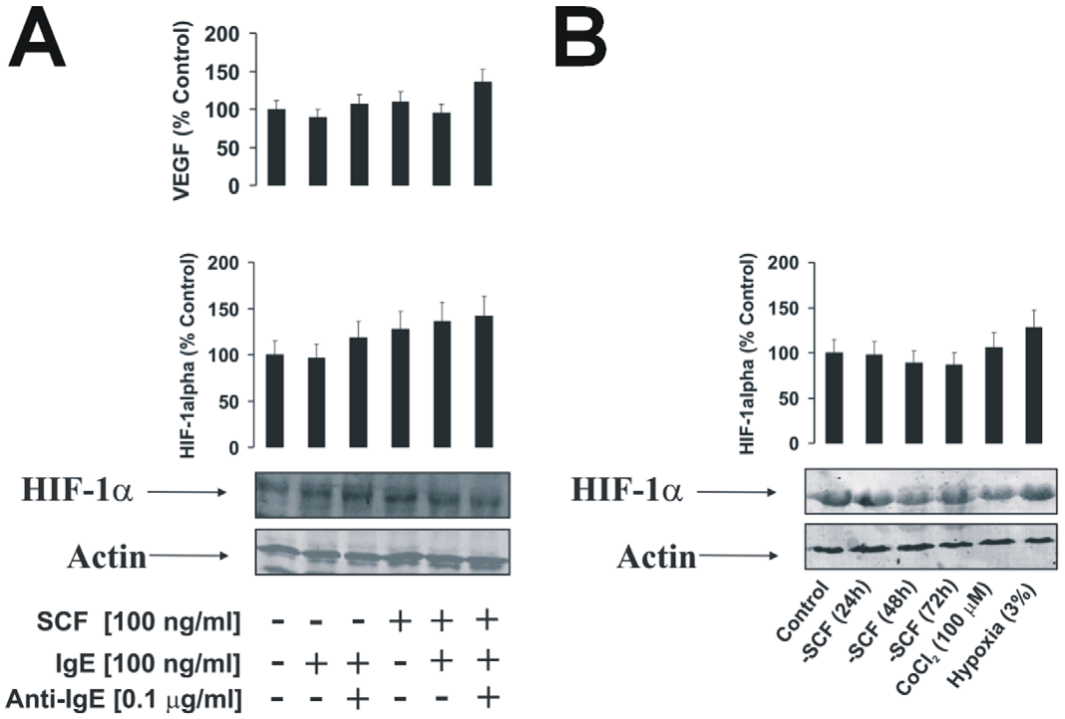
Basic accumulation of HIF-1α protein in LAD2 human mast cells does not depend on the SCF. **A**) Non-sensitised LAD2 cells and those sensitised with 100 ng/ml IgE for 24 h were cultured in the presence or absence of 100 ng/ml SCF. Some of the IgE-sensitised cells were then exposed to 0.1 µg/ml anti-IgE for 2 h. HIF-1α accumulation was then analysed by Western blot. VEGF mRNA levels were analysed by qRT-PCR. **B**) LAD2 cells were cultured in the absence of SCF for 24, 48 and 72 h. In addition, LAD2 cells, cultured as normally in the presence of SCF, were kept 4 h under hypoxic (3% O_2_) conditions or exposed for 4 h to 100 µM cobalt chloride. HIF-1α accumulation was analysed by Western blot. Quantitative data are mean values+S.D. of at least three individual experiments. * indicates p<0.01 vs. control. All Western blot data are from one experiment representative of three that gave similar results.

We discovered that SCF non-significantly upregulated HIF-1α accumulation in the LAD2 cells compared to those cultured in the absence of SCF. In all other cases the levels of HIF-1α were very similar (Figure 6A). We also examined whether long-term SCF withdrawal could lead to a downregulation of HIF-1α accumulation. We therefore cultured LAD2 cells in the absence of SCF for 24, 48 and 72 h. In addition, two positive controls of recognised HIF-1α inducers were used. Here, LAD2 cells were cultured in the presence of SCF but exposed for 4 h either to hypoxic (3% O_2_) conditions or to 100 µM cobalt chloride. We observed, however, that SCF withdrawal did not significantly downregulate HIF-1α accumulation in LAD2 cells, regardless of the period of SCF withdrawal (Figure 6B). Furthermore, neither hypoxia nor cobalt chloride were able to significantly upregulate HIF-1α protein accumulation in LAD2 cells (Figure 6B). SCF, therefore, does not appear to be a major contributor in determining the accumulation of HIF-1α protein in non-stimulated LAD2 mast cells. Importantly, the absence of marked increases in HIF-1α protein accumulation following hypoxia/cobalt chloride treatment suggests that these cells require baseline levels of this protein at near-maximum threshold (those for mRNA levels were reported before [25]).

### HIF-1α protein plays a crucial role in the activity of LAD2 mast cells

**Figure 7:**
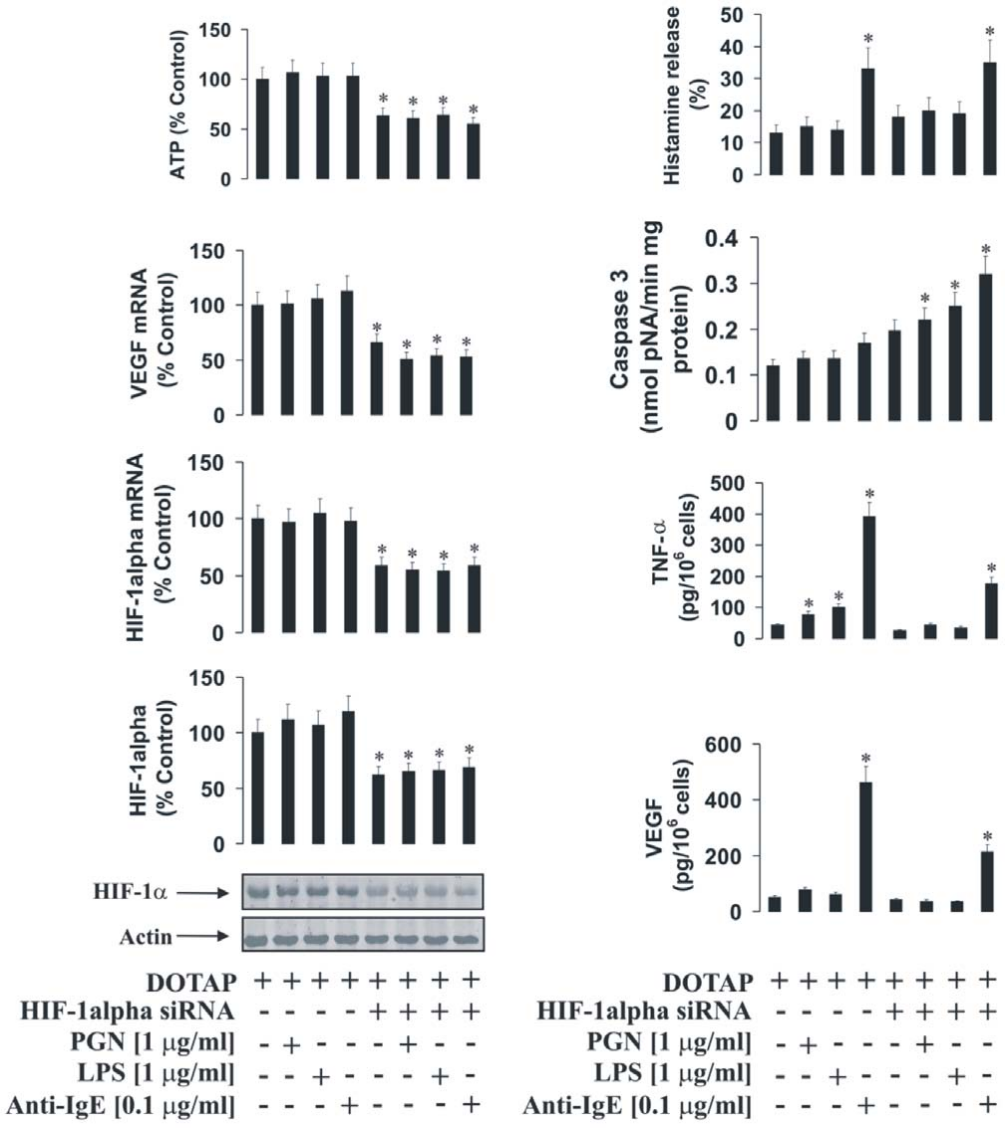
HIF-1α protein plays a pivotal role in the inflammatory responses of LAD2 mast cells. (**A**) Normal and HIF-1α knockdown LAD2 cells were sensitised with 100 ng/ml IgE for 24 h followed by 4 h exposure to 1 µg/ml PGN, LPS or 0.1 µg/ml anti-IgE. HIF-1α accumulation/mRNA levels, VEGF mRNA expression/VEGF release, histamine and TNF-α release, intracellular ATP levels as well as caspase 3 activity were analysed as described in Materials and methods. Quantitative data are mean values+S.D. of at least three individual experiments. * indicates p<0.01 vs. control. All Western blot data are from one experiment representative of three that gave similar results.

**Figure 8:**
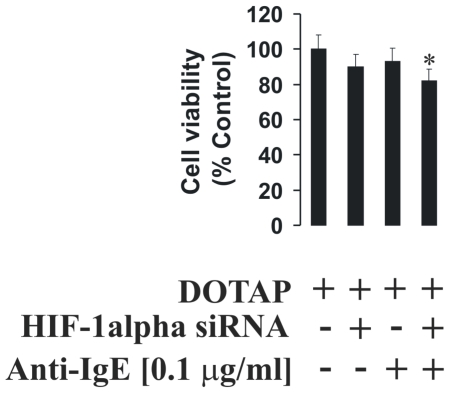
HIF-1α protein supports LAD2 mast cell viability during IgE-induced responses. Normal and HIF-1α knockdown LAD2 cells were sensitised with 100 ng/ml IgE for 24 h followed by 2 h exposure to 0.1 µg/ml anti-IgE (or buffer control). MTS assay was then performed (see Materials and methods). Quantitative data are mean values+S.D. of at least three individual experiments. * indicates p<0.01 vs. control. a – indicates significant (p<0.01) differences between the combined stimulation of basophils with PGN and anti-IgE with PGN or anti-IgE alone.

To characterise the role of the HIF-1 transcription complex in LAD2 cell responses we used normal and HIF-1α knockdown LAD2 cells. The knockdown was achieved by transfection of the cells with HIF-1α siRNA. HIF-1α knockdown was assayed by quantitative real-time PCR (qPCR) and Western blot analysis. Both normal and HIF-1α knockdown LAD2 cells were sensitised with 100 ng/ml IgE and exposed to PGN, LPS or anti-IgE. We found that in both stimulated and non-stimulated HIF-1α knockdown cells, the expression of VEGF was significantly lower and ATP levels were significantly downregulated (Figure 7). TNF-α and VEGF production by LAD2 mast cells were affected by HIF-1α knockdown, which also resulted in increased caspase 3 activity (Figure 7). Only histamine release was not affected in HIF-1α knockdown cells (Figure 7). MTS assay demonstrated a slight, but significant, decrease in cell viability after 2 h of exposure to 0.1 µg/ml anti-IgE (Figure 8) following HIF-1α knockdown in IgE-sensitised LAD2 cells. This observation is consistent with the results demonstrating an increase in caspase 3 activity and ATP depletion (Figure 7).

### Cross-interaction of IgE-mediated and PGN/LPS-mediated responses in primary human basophils and THP-1 myeloid cells

**Figure 9:**
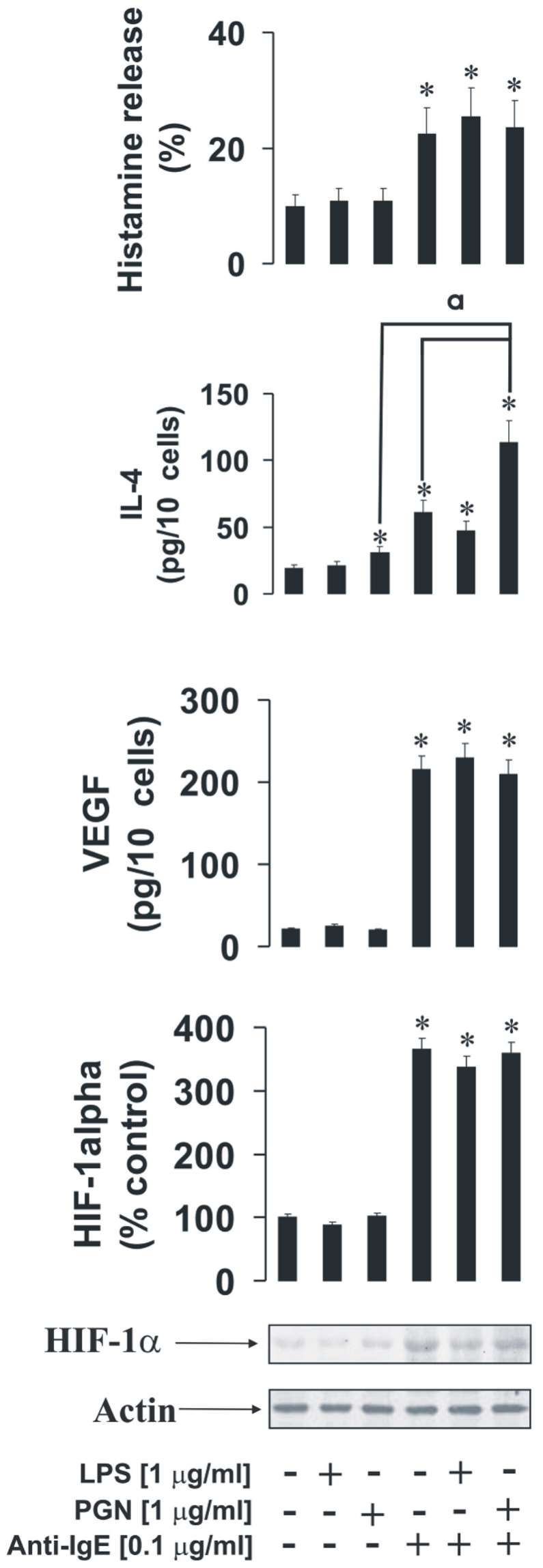
The effects of PGN and LPS on IgE-induced responses of primary human basophils. Primary human basophils were exposed to for 4 h to 1 µg/ml PGN or LPS (some of the samples were left untreated). Some of the cell samples (including TLR ligand treated and untreated) were then exposed to 0.1 µg/ml anti-IgE for 2 h. HIF-1α accumulation, VEGF, histamine and IL-4 release were analysed as described in Materials and methods. Quantitative data are mean values+S.D. of at least three individual experiments. * indicates p<0.01 vs. control. All Western blot data are from one experiment representative of three that gave similar results.

We investigated whether TLRs 2 and 4 are able to induce inflammatory reactions in primary human basophils. We were also interested if these TLR2/4 ligands are able to potentiate IgE/anti-IgE-mediated pro-allergic responses of basophils. For that purpose basophils were exposed for 4 h exposure to 1 µg/ml PGN or LPS (some of the samples were left untreated). After that, some of the cell samples (including TLR ligand treated and untreated) were exposed to 0.1 µg/ml anti-IgE for 2 h. We obesrved that TLR2/4 ligands did not induce HIF-1alpha accumulation or histamine release in basophils. They also did not upregulate VEGF production. PGN (TLR2 ligand) but not LPS did, however, induce IL-4 production in primary human basophils (Figure 9). Anti-IgE induced accumulation of HIF-1α and VEGF production were not potentiated by PGN or LPS. Anti-IgE-induced histamine release was also not further upregulated by TLR2/4 ligands (Figure 9). Anti-IgE-induced IL-4 production was significantly potentiated by PGN but not LPS demonstrating differential responses of basophils and mast cells.

**Figure 10:**
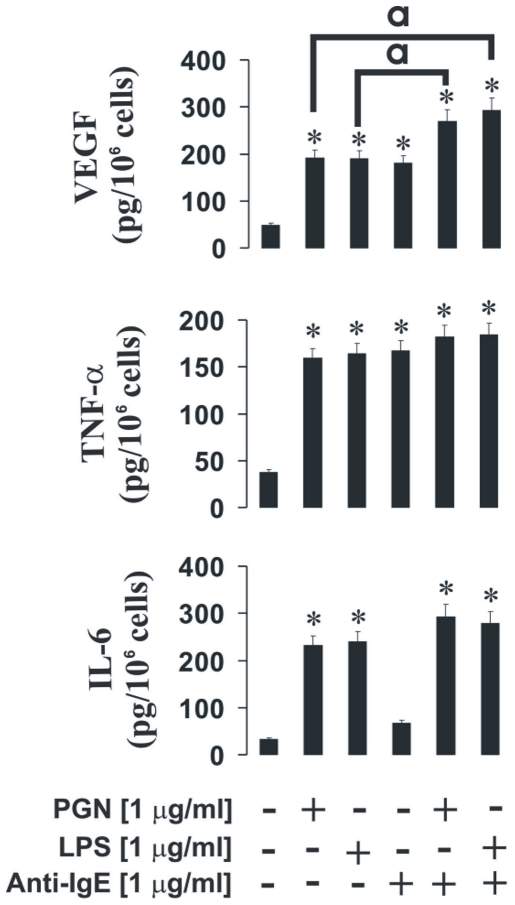
The effects of PGN and LPS on IgE-induced responses of THP-1 human myeloid cells. THP-1 cells were sensitised with 100 ng/ml IgE for 24 h followed by the treatments described above for basophils, except that an anti-IgE concentration of 1 µg/ml was used. Production of VEGF, TNF-α and IL6 was then analysed by ELISA as outlined in Materials and methods. Quantitative data are mean values+S.D. of at least three individual experiments. * indicates p<0.01 vs. control. **a** – differences are significant between indicated values (p<0.01).

Finally, we also assessed the responses of human myeloid THP-1 cells, which express FcεRII IgE receptor (CD23). THP-1 cells were sensitised with 100 ng/ml IgE for 24 h followed by the treatments described above for basophils, but the anti-IgE concentration of 1 µg/ml was used (due to the low affinity of the receptor [14]). THP-1 cells responded to stimulation with anti-IgE by releasing TNF-α, VEGF and small amounts of IL-6. Production of VEGF, but not other cytokines, was potentiated by both PGN and LPS (Figure 10). Our ongoing experiments demonstrate clear induction of HIF-1α accumulation in IgE-sensitised THP-1 cells by all the inflammatory mediators tested (PGN, LPS and anti-IgE – data not shown). PGN and LPS as the inflammatory TLR ligands were able to induce production of IL-6, TNF-α and VEGF (Figure 10). Non-sensitised THP-1 cells showed no responses whatsoever to stimulation with 1 µg/ml anti-IgE (data not shown).

## Discussion

Our studies show that HIF-1 generally plays an important role in supporting the ability of allergic effector cells to generate inflammatory and immunomodulatory cytokines. In contrast, degranulation of mast cells and basophils does not seem to be affected by HIF-1. However, comparing mast cells to basophils and also THP-1 cells we discovered crucial differences in HIF-1 input. Whereas, in basophils, HIF-1 is strikingly induced by IgE-dependent triggers, in mast cells the protein is clearly expressed constitutively but only weakly induced by stimulation. One might have expected this to be due to the presence of SCF in LAD2 mast cell cultures but, surprisingly, LAD2 cells cultured for 24–72 h in the absence of SCF still expressed substantial levels of HIF-1α. Similarly, IgE sensitization only weakly increased constitutive HIF-1α expressions. This supports the notion that mast cells are highly dependent on cytosolic oxygen metabolism [26], [27], which is controlled by HIF-1 complex on the transcriptional level [5]. However, while this is supported in LAD2 mast cell lines, which resemble the general characteristics and functions of primary tissue mast cells more closely than other cell lines, it is important to recognise that primary human mast cells are heterogeneous and may differ in function depending on their tissue location. Importantly, the malignant phenotype of the LAD2 cell line might also influence constitutive HIF-1α, thereby increasing the sensitivity of these cells to its loss. It would therefore be interesting to further investigate the crucial role of HIF-1 in primary mast cells and using *in vivo* models.

It has long been appreciated that mast cells and basophils not only express FcεRI but also TLRs2/4 [9]–[11]. These receptors recognise molecular patterns shared by Gram-positive and Gram-negative bacteria, therefore potentially amplifying the pro-allergic (Th2) responses of these allergic effector cells [1], [2], [9], [10]. However, the cross-interactions between these two systems remain unclear. In the present study we found that LAD2 human mast cells and primary human basophils express detectable amounts of TLR2 and TLR4, although the levels of these receptors in both cell types are much lower compared to those in THP-1 human myeloid cells (Figure 1). IgE-mediated histamine, VEGF and TNF-α releases in mast cells were not affected or potentiated by PGN/Pam3Cys or LPS.

As with IgE-dependent triggering, TLR2/4 ligands (PGN/Pam3Cys and LPS respectively) were unable to induce clear increases in HIF-1α accumulation in LAD2 mast cells. Both ligands, however, upregulated TNF-α production, although much less so compared to THP-1 cells. However, TLR ligands failed to induce VEGF production or histamine release in mast cells. IgE-mediated stimulation in mast cells also failed to significantly upregulate HIF-1α accumulation. This was supported by the lack of increases in IgE-induced VEGF mRNA expression, though VEGF protein was released. These results suggest that the level of constitutive HIF-1α expression may be sufficient to meet the stress adaptation requirements given the fact that the levels of the intracellular ATP were not affected.

Employment of silencing RNA to knock-down HIF-1α expression confirmed that, while HIF-1α is only poorly induced by stimulating mast cells with a variety of secretagogues, it still plays a fundamental role in mast cell survival and activity. HIF-1α knock-down significantly reduced the ability of mast cells to express VEGF and to produce TNF-α in response to stimulation with TLR2/4 or FcεRI ligands, although it had no effect on degranulation. The levels of intracellular ATP were also significantly reduced, which correlated with caspase 3 activation in ligand-stimulated LAD2 cells. The observed decrease in viability of HIF-1α knockdown LAD2 mast cells further indicates that their adaptation to any kind of inflammatory stress or their proper functioning in principle becomes problematic in the absence of this protein and further highlights their reliance on glycolysis. In addition, it is also possible that IgE-dependent histamine releases may be more affected by longer treatment periods with HIF-1α siRNA due to decreased cell viability.

In basophils TLR2/4 ligands were unable to induce significant increases in HIF-1α accumulation on their own whereas anti-IgE induced HIF-1α accumulation, which was consistent with our previously published data [6]. As with mast cells, PGN or LPS failed to induce histamine release in basophils. LPS was unable to induce IL-4 or VEGF release, while PGN induced IL-4 but not VEGF release in basophils and potentiated IgE-induced IL-4 release. In this case one could suggest that TLR2 in basophils displays higher inflammatory activity compared to LAD2 mast cells where TLR4 seems to be more active (Figure 9). Basophil TLR4 did not display any signs of pro-inflammatory activity, which is consistent with recently published observations [9], [10]. PGN-induced IL-4 production in basophils does not seem to recruit HIF-1. This is most likely associated with the fact that PGN does not induce any histamine release and thus energy consuming major alterations of cytoskeleton associated with degranulation do not take place.

Our results show that pro-allergic IgE-dependent responses are not significantly potentiated by TLR2/4 ligands. This suggests that, in cases of bacterial infection in asthmatic airways, where mast cells and basophils may be stimulated by both TLR2/4 ligands and by IgE-dependent mechanisms, it is unlikely that IgE-dependent responses are potentiated by pathogen-associated TLR2/4 ligands [9]–[13].

In THP-1 human myeloid cells anti-IgE stimulation induced the release of TNF-α and VEGF. Slightly increased release of IL-6 was also detected. IgE-dependent stimulation did not lead to a potentiation of TLR2/4-mediated TNF-α or IL-6 release. LPS or PGN-induced VEGF production in THP-1 cells was, however, potentiated by anti-IgE, in stark contrast to mast cells or basophils.

**Figure 11:**
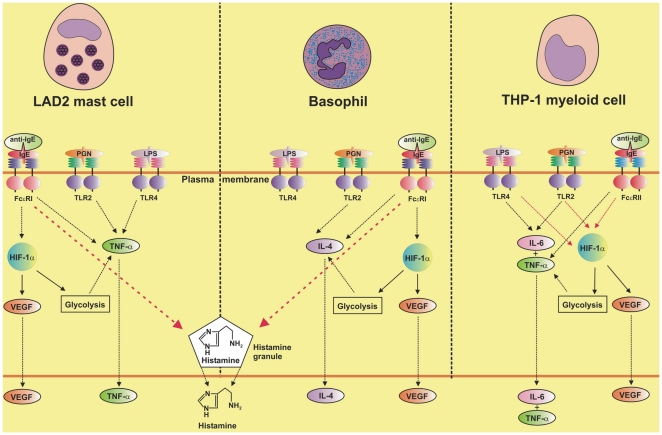
Comparative analysis of HIF-1α involvement in inflammatory responses and their cross-interactions in LAD2 human mast cells, primary human basophils and THP-1 human myeloid cells. This scheme is based on our current findings reported above and also on our previous observations [6], [18].

Taken together our results demonstrate that both LAD2 mast cells and basophils respond differentially to co-stimulation with pro-allergic stimuli and pathogen-derived TLR2/4 ligands. Both cell types do not strongly respond to ligand-induced TLR2/4 activation in comparison to human myeloid innate immune cells. This could be a result of lower levels of expression as well as lower activity of these receptors in mast cells and basophils compared to myeloid cells. The presence of constitutive HIF-1 in LAD2 mast cells was revealed to be essential for them in order to respond to both IgE-dependent and TLR-mediated stimulation. However, in contrast to basophils, increases in HIF-1 expressions to these modes of activation were not pronounced. In basophils the protein also plays an essential role in IgE-dependent activation but less so for TLR2-mediated triggering. The results discussed above are summarised in the scheme presented in the Figure 11.
